# Equity by Design – Using Digital Technology To Overcome Cardiovascular Health Disparities

**DOI:** 10.1007/s11886-025-02319-3

**Published:** 2025-11-27

**Authors:** Priya Nair, Michelle Dai, Dhruvil A. Patel, Kevin Pearlman, Sachin D. Shah

**Affiliations:** Department of Medicine, University of Chicago Medical Center, 5841 S. Maryland Ave, Chicago, IL 60637 USA

**Keywords:** Telehealth, Cardiovascular, Disparities, Equity, Usability, Digital

## Abstract

**Purpose of Review:**

This paper examines how digital health technologies can either reduce or exacerbate disparities in cardiovascular disease outcomes. It explores the role of the digital divide defined as a multifactorial phenomenon encompassing unequal access to internet connectivity, digital devices, and digital literacy, often driven by socioeconomic status, geography, age, education, language, and disability in shaping access to telehealth, remote monitoring, and patient engagement tools, with a focus on strategies for equitable implementation.

**Recent Findings:**

Digital health interventions have improved patient outcomes by enhancing accessibility and adherence to care. However, challenges such as limited technology access, low digital literacy, and systemic biases contribute to persistent disparities, particularly among marginalized populations. Emerging strategies, including community-based digital literacy programs, policy reforms, and inclusive design approaches, show promise in addressing these gaps.

**Summary:**

While digital health has the potential to bridge gaps in cardiovascular care, ensuring equity requires intentional design and policy interventions. Addressing barriers to access and digital literacy is critical to maximizing the benefits of these innovations for all patient populations.

## Introduction

Cardiovascular disease (CVD) remains the leading cause of mortality across most major age groups in the United States, and it disproportionately affects minority populations. Compared to non-Hispanic White adults, non-Hispanic Black adults exhibit higher rates of CVD and associated risk factors, including hypertension, obesity, diabetes, and heart failure. As a result, Black adults continue to experience higher mortality rates compared to White adults despite overall declines in age-adjusted cardiovascular mortality rates between 1999 and 2019. In 2019, the cardiovascular mortality rate was 1.32 times higher for Black women than for White women and 1.33 times higher for Black men than for White men. In addition to race, higher CVD mortality rates are also linked to limited financial resources, rural residency, and residence in regions with greater racial segregation [1].

Between 2009 and 2018, CVD mortality rates declined across most U.S. counties, yet rural counties consistently experienced higher rates compared to urban areas [2]. Today, 86% of rural counties lack a practicing cardiologist, contributing to longer travel for care, worse health outcomes including higher mortality and shorter life expectancy, and compounding disadvantages such as lower incomes, higher uninsured rates, limited access to healthy food and primary care, and more preventable hospitalizations [3]. As a result, rural patients are less likely to receive guideline-based CVD care. Recent evidence further supports this concern: access to cardiology and endocrinology care among adults with type 2 diabetes and atherosclerotic cardiovascular disease is significantly shaped by geographic barriers, with greater distance to specialty clinics reducing the likelihood of receiving care [4]. Although telemedicine expanded access for some patients, disparities persisted by rurality, age, and race, underscoring that digital health alone cannot overcome the geographic and socioeconomic challenges limiting specialty care access [4]. Together, these findings highlight the urgent need for strategies that specifically address the geographic barriers faced by rural populations in accessing cardiovascular care.

The use of digital technologies may offer a promising approach to mitigating health inequities. In recent years, telehealth, remote monitoring, and patient portals have revolutionized how healthcare is delivered by enabling greater accessibility, convenience, and personalization. However, disparities in access to digital resources commonly referred to as “the digital divide” persist. This gap has emerged as a significant barrier to equitable healthcare access, and it disproportionately impacts vulnerable populations such as the elderly, people of color and non-native English speakers while further exacerbating existing health disparities [5].

This paper aims to explore the concept of “Equity by Design” in mitigating health disparities through the strategic integration of digital technology in cardiovascular disease management. Specifically, it will examine the impact of the digital divide on vulnerable populations, assess the promise and potential pitfalls of digital health tools, and present recommendations for advancing digital health solutions in ways that are inclusive, accessible, and ultimately equitable.

## Impact of COVID-19 on the Digital Divide

The COVID-19 pandemic accelerated the U.S. healthcare system’s reliance on digital health technologies. Telehealth visits surged by 154% in March 2020 compared to the same period in 2019 [6], and telemedicine has remained a substantial mode of care even as in-person services returned [7, 8]. At the University of Chicago Medical Center, virtual visits made up 60.5% of ambulatory care between March and May 2020, but stark disparities emerged: older adults, Black patients, and those with public insurance were more likely to have telephone rather than video visits, reflecting national patterns [9–11]. For example, a Veterans Affairs (VA) study found rural veterans were significantly less likely to use video visits than urban veterans, highlighting broadband and device gaps [11]. These disparities are important because patients who use video visits generally report higher satisfaction and receive better-quality care compared to those relying on telephone visits [12, 13].

Barriers to accessing high-quality virtual health care are multifactorial and disproportionately affect vulnerable populations. Limited broadband and reliable connectivity remain foundational barriers, particularly for rural communities, low-income households, and areas affected by digital redlining. These infrastructure gaps reduce video-based telehealth use, portal engagement, and access to advanced virtual care [14]. Low digital literacy further limits participation, especially among older adults, individuals with lower educational attainment, and underrepresented racial and ethnic groups. Beyond technical skills, digital literacy requires confidence and self-efficacy; its absence is a major determinant of telehealth non-use [15]. Lack of home support, such as a caregiver to assist with technology, also restricts access for older adults and those with cognitive or physical impairments, leaving many unable to complete virtual visits [16]. Usability issues, small font sizes, complex navigation, poor compatibility with assistive devices, and limited accommodations exacerbate barriers for older adults, individuals with disabilities, and those with sensory or cognitive impairments. Generic platforms often neglect these needs, worsening exclusion [14]. Codesign approaches with patients and caregivers could address these challenges but remain uncommon [14].

Collectively, these findings underscore that while the rapid expansion of telehealth has increased access to care, persistent structural, technological, and literacy barriers disproportionately limit participation among vulnerable populations. Addressing these challenges through targeted interventions, inclusive design, and supportive infrastructure is essential to ensure digital health fulfills its potential for equitable care.

### The Promise and Perils of Digital Technology in Addressing Cardiovascular Health Disparities

While the COVID-19 pandemic shed light on the deep-rooted digital divide, it also catalyzed a surge in digital health innovations that offer both unprecedented opportunities and new challenges for addressing long-standing cardiovascular disparities. At the Cleveland Clinic, remote blood pressure monitoring programs led to a 20% improvement in medication adherence and a 15% reduction in emergency department visits [17]. A Minnesota-based study showed patients enrolled in home blood pressure telemonitoring achieved better blood pressure control for 12 months than those receiving standard care [18]. Similarly, a 2018 study at Kaiser Permanente found that providing devices and streamlining scheduling increased video visit use by 25% among Black patients and 29% among Hispanic patients [19]. Across modalities, digital technologies have the potential to enable more timely diagnosis and management of high-risk cardiovascular conditions such as unstable angina, atrial fibrillation, and congestive heart failure. A meta-analysis of over 50 randomized controlled trials from 2020 to 2021 found that telehealth increased clinical contact by 43% and reduced time to intervention by an average of 9.3 days for rural CVD patients [20]. These findings underscore how digital tools can bridge geographic and systemic barriers and improve outcomes for remote and underserved patients. Despite these gains, the benefits of digital health have not reached all populations equally. Closing persistent gaps in broadband, devices, and digital skills remains essential to realizing digital health’s full promise for cardiovascular care.

## Implementation Strategies and System-Level Considerations for Equitable Digital Health

### Digital Barriers and Equity Metrics

Recognizing the growing importance of digital access for health equity, health systems must move beyond awareness to action. A recent quality improvement initiative at the Department of Veterans Affairs (VA) found that 42.7% of veterans reported at least one digital barrier, including lack of affordable broadband, missing devices, or limited digital literacy [21]. These gaps were most common among older veterans, people of marginalized social status, lower-income individuals, and those with complex medical conditions [21]. To address these disparities, the VA launched the Digital Divide Consult, connecting patients with devices and affordable internet providing a scalable model for closing digital gaps in care [22]. Clinicians also play a vital role in building trust and digital confidence, particularly among communities that have faced long-standing barriers to technology. Yet, success cannot be measured by engagement alone; equity metrics such as usage by race, income, geography, language, and clinical outcomes like hypertension control and cardiovascular events must guide interventions [22]. Key barriers and examples are summarized in Table 1.

**Table 1:** Key barriers to digital health equity and practical examples

Barrier	Example VA Findings
Broadband access	23.2% lacked consistent or affordable broadband
Device availability	16.9% did not own a smartphone or computer
Digital literacy	12.1% needed help setting up or using devices
Financial constraints	Lower-income patients far less likely to use video visits ^21^
Rural access challenges	Broadband gaps and longer distances for service ^19^
Usability/training needs	Many patients want in-person setup help ^20^

### Financial and Structural Barriers

Financial and structural barriers further compound digital inequities. A study of 905 U.S. cities (2017–2021) found that roughly 30% of households lacked broadband, with availability lowest in low-income and predominantly minority neighborhoods [23]. These gaps are not explained by income alone. Broadband adoption remains significantly lower in historically redlined communities where infrastructure providers systematically underinvest; a phenomenon now termed digital redlining. Even in economically similar cities, formerly redlined neighborhoods lag behind non-redlined counterparts in broadband availability [24]. Remote rural areas and Native American tribal communities also face large broadband gaps, as private providers often lack incentives to serve sparsely populated regions. Sustained public investment and federal policy are essential to bridging these divides and ensuring equitable access to digital health innovations (Table 2).

**Table 2:** Federal programs to expand broadband and reduce digital inequities

Program/Policy	Description
BEAD Program	Broadband Equity, Access, and Deployment program funding large-scale infrastructure projects ^9^
USDA ReConnect	Rural Development Broadband ReConnect Program for underserved rural areas ^9^
Broadband Infrastructure Program	Federal grants for broadband expansion ^9^
FCC Lifeline	Discounted internet services for low-income households ^9^
FCC COVID-19 Telehealth Program	Supported providers to expand telehealth during the pandemic ^10^
ACA EHR Funding	Affordable Care Act funding for EHRs and telehealth adoption at community health centers ^15^
Medicare Flexibilities	Short-term extension (2025) of Medicare telehealth flexibilities through the American Relief Act ^24^
CMS Equal Reimbursement	Pandemic-era policy reimbursing telehealth at parity with in-person visits (expiring 2025) ^25, 26^
Ongoing Policy Needs	Continued legislative action needed to sustain and expand digital health access ^27^

Income-related disparities further exacerbate these structural barriers. Over half (53.1%) of patients with household incomes above $75,000 had a video visit in the past year, compared with only 19.6% of those earning less than $35,000, highlighting financial resources as a key determinant of access to technology, broadband, and digital skills [25, 26]. Higher-income households are more likely to afford devices, reliable high-speed internet, and digital literacy support, whereas lower-income households face barriers across all domains [25, 26]. These factors are critical for equitable virtual care, as access to devices and broadband is a prerequisite for video visits, which are associated with higher-quality clinical interactions and better health outcomes than audio-only visits. The American Academy of Pediatrics notes that financial constraints limit access to hardware and broadband, delaying or preventing adoption of new technologies in under resourced populations [27]. Lower-income patients also often lack private spaces for telehealth, further limiting participation [26]. Without targeted interventions addressing affordability, infrastructure, and digital literacy, virtual care risks exacerbating existing health disparities [28]. Monitoring usage by income and other equity metrics is essential to ensure virtual care narrows, rather than widens, health outcome gaps.

### Geographic and Rural Considerations

In addition to financial barriers, geographic location further shapes digital inequities. Rural and remote populations face unique challenges in access to devices, broadband, and training, which require targeted strategies to ensure equitable participation in virtual care. Rural populations face unique challenges in digital access that also demand targeted solutions. One effective strategy involves providing training programs tailored to local needs. For example, an Australian study of 40 adults in rural areas found that participants reported higher confidence in using digital health literacy programs, as measured by the eHealth Literacy Scale (eHEALS); barriers such as product complexity, mistrust, and cost were mitigated through personalized support [29]. Similar programs in rural India have improved digital empowerment and narrowed gaps in access [29]. In the United States, several initiatives address rural digital inequities: the U.S. Department of Education funds adult training in digital problem-solving [30]; Microsoft Philanthropies, in partnership with the Public Library Association, launched DigitalLead to expand access and training in rural libraries; and the Rural Local Initiatives Support Corporation (LISC) Digital Navigators program, supported by the Ford Foundation and partners, connects residents with discounted internet and one-on-one technical assistance. In 2023, this program served nearly 16,000 clients and supported five broadband projects in Arkansas’s most remote counties [31]. Collectively, these efforts highlight the need for sustained, context-specific investment to ensure rural communities can fully benefit from digital health innovation.

### Impact of Equitable Digital Health on Clinical Outcomes

When structural and implementation barriers are addressed, digital health interventions can yield measurable improvements in clinical outcomes, particularly in hypertension control. A systematic review and meta-analysis of 28 studies involving underserved populations found that digital health interventions most commonly remote blood pressure monitoring combined with tailored support resulted in statistically significant reductions in systolic blood pressure at 6 and 12 months (mean difference 4.24 mm Hg and 4.30 mm Hg, respectively) compared to standard care [32]. Similarly, the DIG IT initiative, which provided digital tools alongside interprofessional team support for medically underserved patients, achieved a twofold greater reduction in both systolic BP and 10-year ASCVD risk scores compared to matched controls receiving usual care [33].

These findings underscore that equitable access to high-quality internet, devices, and digital literacy support is essential for optimizing preventive cardiovascular care and reducing disparities in outcomes such as hypertension and cardiovascular disease events [34, 35]. Community-engaged digital infrastructure initiatives, combined with culturally tailored interventions and ongoing support, demonstrate that digital health can be both clinically effective.

### Ethical Co-Design

In this context, moving beyond digital literacy to ethical co-design is critical for ensuring that digital health tools are not only functional but also equitable. Ethical co-design emphasizes transparency, accountability, and authentic community partnership in developing digital health interventions, ensuring equity, inclusivity, and harm reduction while mitigating the risk of reinforcing existing health inequities. Participatory Design (PD), originating in political decision-making and labor rights movements, has gained traction as a methodological approach for democratizing design processes [36]. Increasingly, PD has been leveraged to engage marginalized populations, with the dual goals of fostering empowerment and ensuring that digital tools reflect community priorities [36]. In the U.S. context, underserved groups include low-income, older, queer, trans, gender-nonconforming, ethnic, disabled, and racialized populations, all of whom face structural barriers to technology access and engagement.

Despite its promise, critiques of PD highlight challenges in implementation and framing, noting that poorly executed initiatives can strain relationships between researchers and communities and inadvertently reproduce inequities. Frameworks such as the Design Tension Framework illustrate that PD is not merely a method for problem-solving, but a process of negotiating competing goals and values, offering opportunities for reflection and reconstruction of design practices for underserved populations [36]. When conducted inclusively, participatory approaches have demonstrated measurable benefits, including increased knowledge, confidence, and engagement. For example, the FAITH! App, co-created with sixteen African American churches in Minnesota, improved cardiovascular knowledge and self-efficacy in blood pressure management between 2019 and 2020 [37]. These findings underscore the importance of embedding ethics and inclusivity into co-design efforts, ensuring digital health tools are culturally relevant, trustworthy, and effective in reducing disparities [19].

#### Digital Navigators as Catalysts for Digital Inclusion

Among ongoing digital inclusion efforts, digital navigators have emerged as vital contributors to ensuring individuals can access and effectively use digital health resources. These navigators, often trusted community members, play a critical role in connecting patients to internet services, building digital literacy skills, and troubleshooting technology challenges directly at the point of need. From May 2021 to November 2022, the Massachusetts General Brigham (MGB) patient navigator program successfully enrolled 61% of the 13,413 patients it reached, driving significant increases in patient portal enrollment across clinics, particularly among bilingual and Hispanic patients [35]. Similarly, a Boston primary care pilot focused on patients with type II diabetes demonstrated how an integrated digital navigator model can reduce disparities. Through this initiative, 121 patients (31% of the target group) were newly enrolled in the portal, with a majority of enrollees identifying as Black (62%) or Hispanic/Latinx (19%). Portal enrollment increased for Black patients from 49% to 61% and for Hispanic/Latinx patients from 30% to 42% [36].

These findings underscore that digital tools alone are insufficient to close equity gaps. Vulnerable populations often lack the skills, confidence, or trust to engage effectively, so simply providing technology does not ensure use. Digital navigators serve as the dedicated, in-person support that helps patients enroll, learn, and sustain use of digital health tools. By embedding navigators within care teams, health systems can bridge access gaps, build trust, and translate digital innovations into equity and health gains.

#### Inclusive Design as a Prerequisite for Digital Equity

While access to broadband and guidance from digital navigators are critical first steps, they are not sufficient to ensure equitable participation in digital health. True digital equity also requires that platforms themselves be designed for inclusivity and usability. A 2023 systematic review found that fewer than half of 95 cardiovascular mobile applications met basic accessibility standards, and only 17% offered bilingual interfaces [14]. Similarly, in focus groups of more than 4,000 participants, nearly half felt that telehealth limited their ability to communicate effectively, with Indigenous participants reporting especially strong concerns about cultural alignment and care quality [12]. Accessibility barriers are also pronounced among patients with disabilities: during the pandemic, more than half of visually impaired patients encountered difficulties using telehealth [13].

The American Heart Association (AHA) has recommended embedding inclusive features such as translation services, voice activation, and captioning directly into digital health platforms [19]. Programs like Atlanta’s Inspired initiative demonstrate the importance of linguistic inclusion, pairing digital translation services with in-person support to advance digital literacy. By 2024, this initiative had reached 2,918 participants through more than 150 multilingual workshops [21, 29–41]. Likewise, the iMHere 2.0 mobile health system for patients with cerebral palsy and spinal cord injuries improved usability by integrating customizable features tailored to users with fine motor impairments [42].

These examples illustrate that digital inclusion cannot be achieved through access and training alone. Platforms must be co-designed with marginalized communities to ensure cultural, linguistic, and functional accessibility. Ethical co-design principles emphasizing shared decision-making, cultural relevance, and attention to power imbalances are essential for moving beyond one-size-fits-all approaches and meaningfully reducing disparities [5].

#### Tailoring Digital Health Interventions To Individual Patient Needs

Personalized care is essential to making digital health interventions effective, especially for minority and underserved patients. Digital needs assessments should be incorporated into clinical workflows to align tools with patients’ actual access, preferences, and comfort with technology. Currently, only 12% of U.S. adults have the health literacy skills required to confidently navigate digital platforms [14]. Recognizing that not all patients can adopt high-tech tools, low-tech modalities such as short message service (SMS) text messaging and audio-only phone visits remain critical, particularly for older adults, rural populations, and patients with low digital literacy [43].

Evidence supports the effectiveness of SMS-based interventions across multiple domains. In diabetes care, SMS reminders improved medication adherence and glycemic control, with one randomized trial showing a 0.5% greater reduction in HbA1c compared to usual care [44]. Meta-analyses have also demonstrated significant improvements in smoking cessation (relative risk of quitting: 1.67, 95% CI 1.46–1.90) and weight loss (mean difference: 1.44 kg, 95% CI 2.03 to − 0.84) with SMS interventions compared to controls [43]. These outcomes suggest that even low-tech tools can meaningfully impact health behaviors and disease management.

While prior sections emphasized the advantages of video visits, audio-only care serves a complementary role rather than a direct substitute. A qualitative study found that patients and providers viewed phone calls as a “vital back-up” or standalone solution for brief follow-ups or when visual assessment was unnecessary [45]. For some patients, particularly those with limited internet access or comfort with technology, basic phone calls or texts feel more accessible and realistic than more complex tools.

To prevent digital innovation from exacerbating inequities, future policy must ensure that flexible, patient-centered modalities are supported. This includes reimbursement for SMS- and audio-based care, systematic screening for digital needs, and pathways to help patients progress toward advanced tools with the support of navigators.

#### The Role of Artificial Intelligence in Advancing Health Equity

Equitable access to artificial intelligence (AI) can help personalize care and reduce disparities. Beyond automating digital literacy training and guiding patients through complex health applications, AI has the capacity to deliver tailored interventions for underserved populations [46]. Clinically, it can flag individuals at risk for cardiovascular disease, predict severity earlier, and analyze large datasets to uncover patterns shaped by environmental and social determinants insights that translate into actionable strategies for prevention and management [47, 48].

However, risks remain. Many models function as “black boxes,” obscuring decision-making and allowing errors or biases to perpetuate inequities [49]. Large-scale AI development also carries environmental costs that fall disproportionately on marginalized communities. To mitigate these harms, equity-focused AI must prioritize explainability and fairness, ensuring outputs are interpretable and free from bias. One promising approach uses explainable AI methods, such as Shapley Additive Explanations (SHAP), which assign each input (e.g., blood pressure, cholesterol, smoking status) a measurable contribution to the overall prediction, helping clinicians understand not just what the model predicts but why [49].

Ethical AI development therefore requires clear definitions of fairness, explicit disclosure of assumptions and goals, and systematic evaluation of equity impacts throughout implementation [50]. Multidisciplinary oversight and genuine community partnership are essential to ensure AI advances health equity rather than undermines it.

#### Further Directions and Recommendation

Ongoing research is essential to understand how digital health interventions impact health equity across diverse populations. Future studies should examine long-term outcomes, including patient engagement, clinical results, cost-effectiveness, and satisfaction, while capturing disparities across income, age, geography, and racial/ethnic groups [51–53]. Real-world data are critical to determining whether digital tools are equitably benefiting all patients or inadvertently creating new barriers. Targeted technology needs assessments prior to implementation can identify specific challenges and preferences in each community, guiding the design, deployment, and evaluation of solutions that align with local realities and patient priorities.

Implementation of digital health interventions must go hand-in-hand with intentional monitoring. Without systematic evaluation, even well-designed tools risk reinforcing existing inequities. Continuous assessment and iterative refinement allow researchers and developers to adapt interventions to improve inclusivity, accessibility, and effectiveness at scale. Equity-focused frameworks should embed patient and provider feedback, ensuring digital tools remain relevant and responsive over time.

**Fig. 1:**
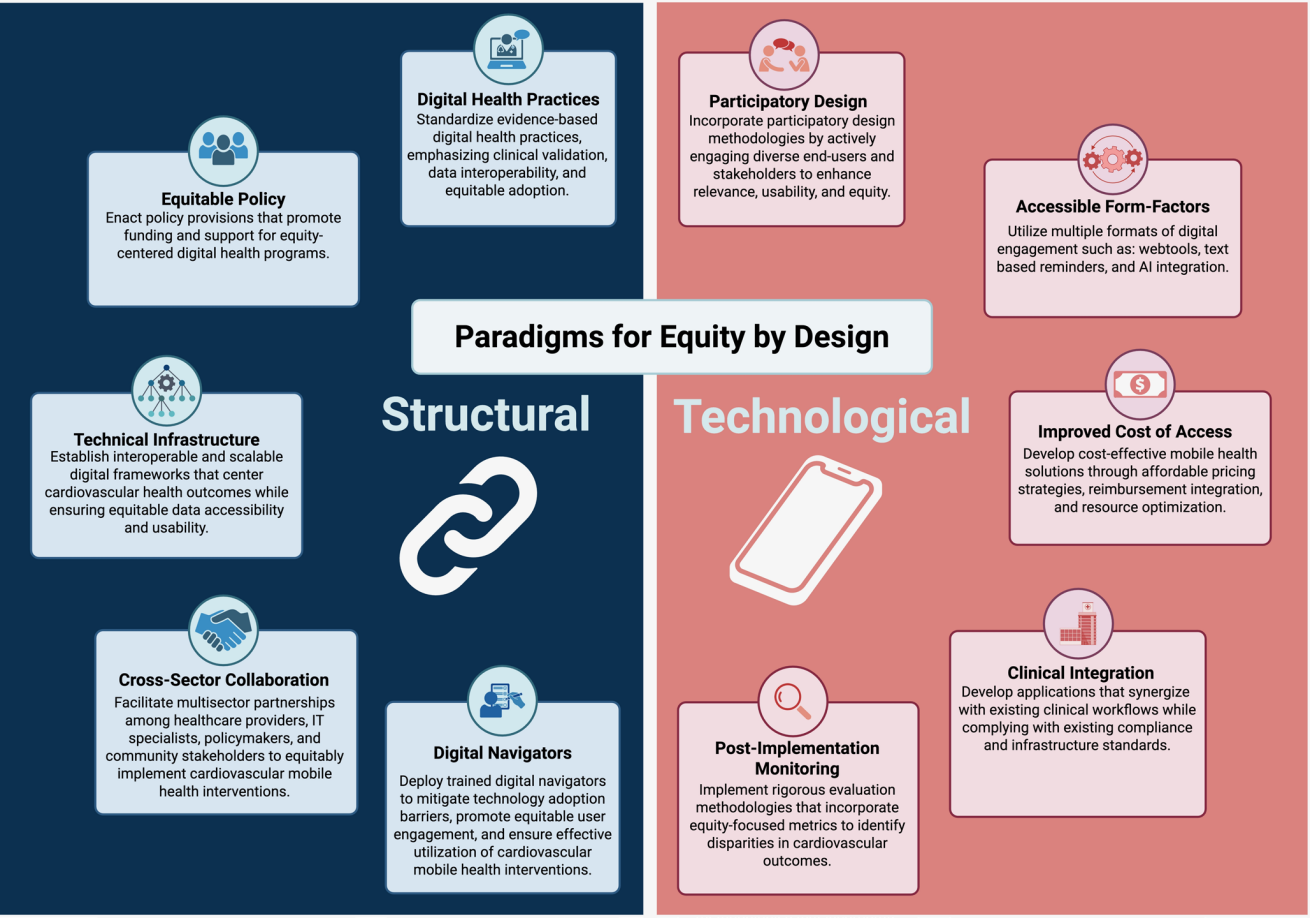
Paradigms for Equity by Design in Digital Cardiovascular Health. This framework illustrates key structural and technological paradigms that support equitable digital health implementation. Structural components include equitable policy, technical infrastructure, cross-sector collaboration, digital navigators, and standardized digital health practices. Technological elements emphasize participatory design, accessible form-factors, cost-effective access, post-implementation monitoring, and clinical integration. Together, these strategies aim to reduce disparities and promote cardiovascular health equity through inclusive and sustainable digital interventions (50). Key for Abbreviations: AI: artificial intelligence; IT: information technology. (Created in BioRender. Patel, D. (2025) https://BioRender.com/fcg541e)

Achieving digital equity requires coordinated action beyond healthcare systems. Technology developers, funders, and policymakers must prioritize underserved communities through sustained partnerships rooted in trust and shared goals. Policies that expand broadband access, provide equitable reimbursement, and support training for digital navigation are essential. Patient-centered, equity-by-design approaches including multilingual interfaces, accessible platforms, and culturally tailored support can ensure that advancements in technology translate into meaningful improvements in health outcomes. Aligning these efforts across stakeholders will help digital health serve as a catalyst for equity rather than a driver of the existing digital divide. These recommendations are summarized in Fig. 1.

## Conclusion

While digital health technologies hold tremendous promise for transforming cardiovascular care and narrowing health disparities, their benefits remain unevenly distributed. Vulnerable populations including people of marginalized social status, low-income individuals, older adults, and rural residents still face barriers such as limited internet access, low digital literacy, and deep-rooted mistrust in the healthcare system. To prevent digital innovation from widening these divides, equity must be embedded from the outset. System-level actions like broadband expansion, regulatory safeguards, and inclusive technology design are critical to ensuring that digital tools reach everyone. At the clinician level, routine digital needs screening, culturally responsive digital navigator support, and patient-centered flexibility including low-tech options can help bridge access gaps in daily care. Equity must not be an afterthought but the foundation of all digital health strategies. With sustained policy investment, interdisciplinary partnerships, and strong accountability, digital health can evolve from a promising tool into a vehicle for justice, ensuring that every patient, regardless of background or income, benefits from advances in cardiovascular care.

## Key References

- Son H, Zhang D, Shen Y, et al. Social Determinants of Cardiovascular Health: A Longitudinal Analysis of Cardiovascular Disease Mortality in US Counties From 2009 to 2018. Journal of the American Heart Association. 2023;12(2). doi:10.1161/jaha.122.026940.
  - Findings from this study suggest rural-urban status, race, income, food, and housing contribute to persistent cardiovascular disease mortality disparities in U.S. counties.
- Lyles CR, Nguyen OK, Khoong EC, Aguilera A, Sarkar U. Multilevel Determinants of Digital Health Equity: A Literature Synthesis to Advance the Field. Annu Rev Public Health. 2023;44:383–405. doi:10.1146/annurev-publhealth-071521-023913.
  - Findings from this study suggest the need for a multilevel approach to digital health equity, highlighting key interventions and recommending steps to improve policy, practice, and research to reduce health disparities.
- Shah SD. From Innovation to Inclusion—Tackling Digital Equity Needs in Health Care. JAMA Network Open. 2024;7(11):e2445334-e2445334. doi:10.1001/jamanetworkopen.2024.45334.
  - Findings from this study suggest bridging the digital divide to ensure equitable access to digital health tools.

## Data Availability

No datasets were generated or analysed during the current study.
